# Characteristics, Outcomes, and Associations of Venous Thromboembolism in Diabetic Patients Infected With COVID-19 in Riyadh, Saudi Arabia

**DOI:** 10.7759/cureus.59468

**Published:** 2024-05-01

**Authors:** Mansour S Albabtain, Khalid A Alyousef, Ziad M Alharbi, Mohammed N Almutairi, Dunia Jawdat

**Affiliations:** 1 College of Medicine, King Saud Bin Abdulaziz University for Health Sciences, Riyadh, SAU; 2 Cellular Therapy Services, King Abdullah International Medical Research Center, Riyadh, SAU

**Keywords:** coronavirus disease 2019 (covid-19), risk factor, electrolytes, diabetes mellitus (dm), venous thromboembolism (vte)

## Abstract

Background

The associations and risk factors for venous thromboembolism (VTE) among hospitalized COVID-19 patients remain ambiguous in the literature, with some conflicting findings, especially in Saudi Arabia. In this study, we aim to elaborate on these data by examining regional patient populations and exploring the incidence, lab findings, and outcomes of VTE among hospitalized COVID-19 patients known to have diabetes mellitus (DM).

Methodology

This cross-sectional study was conducted at King Abdulaziz Medical City in Riyadh. The BestCare system was used to collect patients’ data between September 2020 and February 2022. JMP15 was used for data analysis. Frequencies and percentages were used for categorical data, and median and interquartile ranges were used for quantitative data. The chi-square and Kruskal-Wallis rank-sum tests were used to assess the difference between categorical and quantitative variables, respectively. Nominal logistical regression was used to assess diabetes as a risk factor for developing VTE among COVID-19 patients.

Results

Data from 153 admitted patients were collected after they satisfied the inclusion criteria. Of these patients, 39 (25.49%) developed VTE. The demographic data included age group, gender, and DM status presented as frequencies and percentages. Through bivariate analysis, patients with longer hospital stays had at least one episode of VTE (p = 0.0072). Using nominal logistic regression analysis, diabetes as a risk factor (odds ratio = 4.11, confidence interval = 0.955-5.05, p = 0.0287) was significantly associated with the development of VTE in COVID-19 patients.

Conclusions

Based on our study, diabetes proved significant when evaluating the possible factors regarding VTE development in COVID-19 patients. In addition, the length of stay also played a critical role in the severity of VTE in COVID-19 patients. Similar studies should be conducted on a national scale in Saudi Arabia to accomplish two goals: first, to gain further understanding of the impact of the variables investigated in our population, and second, to publish data that are more generalizable to the larger population of Saudi Arabia.

## Introduction

The severe acute respiratory syndrome coronavirus 2 (SARS-CoV-2) has overwhelmed all facets of the healthcare industry [1]. Wuhan, China, was the first to report the coronavirus disease 2019 (COVID-19) outbreak in December 2019 [2]. The SARS-CoV-2 is an enveloped, positive-sense ssRNA virus classified as a member of the Coronaviridae family and Beta-coronavirus genus [3,4]. Initially seen as a strictly respiratory infection, COVID-19 is now considered a systemic disease as it impacts the respiratory, cardiovascular, hematopoietic, and immune systems, among others [5]. Furthermore, thromboembolic complications are frequently reported in intensive care unit (ICU) and hospitalized COVID-19 patients [6]. The reason behind the incidence of venous thromboembolism (VTE) in COVID-19 patients is yet to be understood [7]. Recent studies found that VTE risk may be as high as 85% despite receiving pharmacological thromboprophylaxis [8].

COVID-19 patients with poor outcomes are characterized by having a higher VTE incidence associated with COVID-19 infection, which includes venous thrombosis, pulmonary embolism, and multiorgan failure [9]. Researchers globally have been assessing blood-borne factors as predictors and prognostic factors related to COVID-19 [1-3,5,7,9-11].

Diabetes mellitus (DM) is a pressing health concern in the 21st century, with the number of patients rising globally in both developed and developing countries. The absence of beta cells or insufficient insulin synthesis by the pancreas are contributing factors to this chronic, complex illness. Diabetes places a substantial burden on patients, families, and healthcare organizations due to its chronic nature, complexity, and expensive treatment. As of 2019, DM has impacted 463 million people [12]. While it is still unclear whether DM patients are more susceptible to COVID-19, numerous studies have found a link between severe COVID-19 cases and DM [3,13,14]. Jarrar et al. conducted a thorough analysis of the literature in 2023, which found a combined prevalence of 16.4% from 2000 to 2020. Additionally, they identified gaps in the existing research on DM in Saudi Arabia, highlighting the need for additional investigations to address these deficiencies [13].

Diabetes in Saudi Arabia is of significant concern, with the country ranking second in the Middle East and seventh in the world, according to the World Health Organization. The prevalence of this chronic disease is on the rise in Saudi Arabia, where an estimated seven million people have diabetes and three million have pre-diabetes. The prevalence of diabetes has increased dramatically nearly tenfold. DM is linked to a host of health problems that impair quality of life, such as increased mortality, morbidity, and vascular complications. Effective preventative and management measures are urgently needed as DM is quickly becoming the top cause of medical complications and deaths in Saudi Arabia [14].

Several biomarkers can be very beneficial as prognostic factors in COVID-19 patients by describing the possible risk of VTE or indicating a superinfection with COVID-19 [2,3,5,9,11,15]. Previous studies have reported dysregulations in biomarkers, including D-dimer, C-reactive protein (CRP), and coagulation factors. Other hematological dysregulation such as thrombocytopenia and lymphocytopenia have also been reported [16].

In recent studies, it has been noted that the increased levels of some biomarkers had a higher association with COVID-19 complications than others. These biomarkers included D-dimer that, when elevated, is associated with coagulopathies and thrombus formation [5,17]. In a previous study, it was noted that D-dimer levels were increased in patients who had the moderate-to-severe form of SARS-CoV-2 infection [10]. Increased levels of D-dimer have also shown an increased in-hospital mortality rate [9].

In brief, with all the literature reviewed, a confirmatory association has not been determined concerning the incidence of thrombotic complications and the severity of the infection caused by SARS-CoV-2 and its use as a prognostic factor [1-12,15-20]. This study aims to evaluate the lab findings regarding VTE incidence in COVID-19 patients and discuss the severity of VTE concerning DM in COVID-19 patients and associated outcomes.

## Materials and methods

Study design, sampling technique, population, and inclusion and exclusion criteria

Admitted patients’ records from King Abdulaziz Medical City (KAMC) in Riyadh in the National Guard Health Affairs (NGHA) were accessed and collected from September 2020 until February 2022 through the BestCare system. As we utilized convenient sampling, all patients who satisfied our inclusion criteria were included in the study.

Our inclusion criteria consisted of diabetic males and females infected with SARS-CoV-2 between the ages of 18-65 years who tested positive on reverse transcription polymerase chain reaction testing. Patients outside the age range, those admitted for fewer than three days, those admitted for a cause other than COVID-19, or those who acquired COVID-19 during their hospital stay were excluded.

All patients were divided into the following three groups based on VTE episodes: null (no episodes), single (one episode), or multiple (two or more episodes). After categorizing patients, qualitative and laboratory data from the patients’ charts were collected at the time of admission, and each patient’s outcome was recorded (e.g., discharge, death, referral outside of the hospital, etc.).

Data collection and statistical analysis

The first set of variables collected from the BestCare system were coagulation factors consisting of D-dimer, activated partial thromboplastin time (aPTT), prothrombin time (PT), fibrinogen, and international normalized ratio (INR). The inflammatory factors including CRP, erythrocyte sedimentation rate (ESR), lactate dehydrogenase (LDH), procalcitonin (PCT), organ function tests of the liver and kidney, as well as electrolytes such as Na^+^, K^+^, and Ca^+2^ were collected.

For descriptive statistics, all outcome variables were represented as mean ± SD if the variables were symmetrically distributed. If the data were skewed or contained significant outliers, median and interquartile range (IQR) were used. Categorical outcome variables were represented as frequencies and percentages.

Regarding inferential statistics, our data were represented with measures of center such as mean and median. The mean or median of each variable (e.g., D-dimer) was taken from each group. Then, analysis of variance (ANOVA) or Kruskal-Wallis rank-sum test was used to determine any statistically significant difference between them. In case ANOVA or Kruskal-Wallis was significant for that variable, Tukey’s post hoc or Steel-Dwass post hoc test was applied to the significantly different variables to determine the magnitude in which that variable was different (i.e., lower or higher than the other means/medians for that variable).

For our study, a p-value <0.05 was considered significant. Microsoft Excel was utilized for data entry, and JMP15 was used for analysis.

Ethical considerations and data management

Institutional review board (IRB) approval was obtained on July 1, 2021 (approval number: SP21R/278/05) to proceed with data collection and analysis. Given that our proposed study concept relied on events that had already occurred between 2020 and 2022, adopting a cross-sectional study design allowed us to assess the exposure to the virus (SARS-CoV-2) and the primary outcome of increased risk of developing VTE and any associated disturbances in biochemical markers in DM patients with COVID-19. Given the design of our observational study, informed consent was not required. With IRB approval and access to hospital databases, all identification data and any other sensitive information relating to the patients, such as names and MRNs, were not collected to ensure confidentiality. Both soft and hard copies were made with backup versions. All editions and updates were dated on each copy and were entrusted to the principal investigator for safekeeping.

## Results

A chart review was conducted on patients admitted to KAMC from September 2020 to February 2022. A total of 596 patients were admitted for COVID-19 and were filtered using our specified criteria. In total, 153 of these patients met our inclusion criteria. They were sampled by non-probability convenience sampling, as shown in Table 1. There were 77 (50.33%) males and 76 (49.67%) females. Of the 153 admitted COVID-19 patients, 39 (25.49%) developed VTE; however, there was no significant association between genders in terms of developing VTE (p = 0.1035). However, there was a significant association between age groups and the development of VTE (p = 0.0363). Moreover, risk factors such as diabetes showed a significant association with VTE development in admitted COVID-19 patients (p = 0.0094), as shown in Table 1. Regarding outcomes, there were no significant associations between VTE development and being discharged, referred, or dying (p = 0.1126), as shown in Table 1.

**Table 1:** Demographic data and VTE development across different age groups, genders, diabetes risk factors, and outcomes. A p-value <0.05 is considered significant. VTE = venous thromboembolism

Did the patient develop VTE? (yes/no) (n = 153)					
	Yes (n = 39) (25.49%)	No (n = 114) (74.51%)	Count	% of total	P-value
Age group (year)					
18–29	0	10	10	6.54%	0.0363*
30–49	10	33	43	28.10%	
50–65	29	71	100	65.36%	
Gender					
Male	24	53	77	50.33%	0.1035
Female	15	61	76	49.67%	
Diabetes					
Yes	34	76	110	71.90%	0.0094*
No	5	38	43	28.10%	
Outcome					
Discharged home	27	96	123	80.39%	0.1126
Referred	2	5	7	4.58%	
Deceased	10	13	23	15.03%	

The overall median length of stay for all patients was 11 days (IQR = 17 days, p < 0.05). Patients who did not develop any VTE episodes stayed significantly less than patients in either the one or two VTE events groups (p < 0.05), nevertheless, the one and two VTE events groups did not differ significantly in terms of hospital stay length (p = 0.1040). The coagulation factors (D-dimer, aPTT, PT, fibrinogen, and INR) did not differ significantly between all VTE groups (p = 0.69, 0.14, 0.153, 0.906, and 0.146, respectively). Furthermore, the liver function parameters (bilirubin, aspartate aminotransferase (AST), and alanine transaminase (ALT)) also did not differ significantly among all VTE groups (p = 0.3606, 0.9666, and 0.1111, respectively). Additionally, electrolytes such as sodium, potassium, and calcium did not differ significantly between the VTE groups, as shown in Table 2.

**Table 2:** Various biomarkers and length of stay between different groups of VTE populations. A p-value <0.05 is considered significant. Medians in each column followed by the same letter are not statistically different (p < 0.05) according to the Steel-Dwass post hoc test. LDH = lactate dehydrogenase; PCT = procalcitonin; BUN = blood urea nitrogen; ALT = alanine transaminase; AST = aspartate aminotransferase; ESR = erythrocyte sedimentation rate; CRP = C-reactive protein; INR = international normalized ratio; PT = prothrombin time; aPTT = activated partial thromboplastin time; VTE = venous thromboembolism; CI = confidence interval

	Number of episodes of VTE (n = 153)					
	No VTE	One VTE	Two or more VTE	Total		
	Median (IQR)	Median (IQR)	Median (IQR)	Median (IQR)	P-value	(95% CI)
Length of stay (days)	10 (12)	21 (22.5)	29 (38)	11 (17)	0.0072*	10-14
D-dimer (ng/mL)	1.005 (1.885)	1.02 (1.02)	1.97 (1.28)	1.035 (1.635)	0.69	Insufficient data
aPTT (seconds)	29.3 (4.87)	28.6 (6.45)	33.85 (14.77)	29.25 (5.625)	0.14	29.2-39.12
PT (seconds)	11 (1.5)	10.95 (1.325)	12.85	11 (1.4)	0.153	11-11.6
Fibrinogen (g/L)	4.46 (3.025)	4.95 (1.38)	Missing (null)	4.46 (2.48)	0.906	Insufficient data
INR	1 (0.15)	1 (0.1225)	1.185 (1.665)	1 (0.15)	0.146	1-1.06
CRP (mg/L)	85.5 (110.25)	117 (171)	56 (109.75)	94 (122.5)	0.375	Insufficient data
ESR (mm/hour)	50 (36.5)	57 (40.5)	62 (0)	54 (25)	0.528	Insufficient data
LDH (U/L)	394.5 (224)	522 (231)	285.5 (235.25)	406 (233)	0.0535	Insufficient data
Ferritin (µg/L)	344.5 (638.95)	714.1 (1755.85)	1075.6 (304.2)	397 (865)	0.1775	Insufficient data
PCT (ng/mL)	0.17 (0.5825)	0.24 (0.6)	1.67 (48.06)	0.215 (0.855)	0.0926	Insufficient data
Creatinine (μmol/L)	87 (140)	92 (168.5)	244 (239)	90 (154.25)	0.6720	81-105
BUN (mmol/L)	6.85 (7.075)	5.1 (10.825)	6.2 (28.1)	6.5 (8.1)	0.7765	5.5-7.6
Bilirubin (μmol/L)	7.8 (6.3)	7.4 (6.7)	12.4 (10.85)	7.85 (6.225)	0.3606	7.7-10.4
AST (U/L)	34 (35)	35 (20.5)	40 (51.5)	35 (27.5)	0.9666	34-41
ALT (U/L)	28.5 (25.25)	22 (19)	30 (64.5)	26.5 (25.75)	0.1111	25-37
Na+ (mmol/L)	136 (5)	133.5 (6.25)	136 (2)	136 (6)	0.0638	135-136
K+ (mmol/L)	4.3 (0.8)	4.4 (0.95)	4.4 (1.2)	4.3 (0.9)	0.0759	4.2-4.4
Ca^2+^ (mmol/L)	2.125 (0.2075)	2.18 (0.23)	2.16 (0.37)	2.14 (0.2)	0.0778	2.1-2.17

Inflammatory markers (CRP and ESR) did not differ significantly between all VTE groups (p = 0.375 and 0.528, respectively). Similarly, kidney function parameters (creatinine and blood urea nitrogen (BUN)) did not differ among all patient VTE groups (p = 0.6720 and 0.7765, respectively), as shown in Table 2. For the miscellaneous parameters (LDH, ferritin, and PCT), there was no significant difference between the patient VTE groups (p = 0.0535. 0.1775, and 0.0926, respectively).

We found that 116 patients (75.82% of the sample) experienced no VTE events, 30 (19.61% of the sample) experienced one VTE event, and five (3% of the sample) experienced two or more VTE episodes. There was statistical significance between the development of VTE and the pre-existence of DM in admitted patients (p = 0.0287) with an odds ratio (OR) of 4.11 of developing at least one VTE episode with a pre-existing diabetes diagnosis (confidence interval (CI) = 0.95-5.05, p = 0.0124), as shown in Table 3.

**Table 3:** Risk factors and outcomes across VTE groups. A p-value <0.05 is considered significant. VTE = venous thromboembolism; OR = odds ratio; CI = confidence interval

	Number of episodes of VTE (n = 153)					
	No VTE	One VTE	Two or more VTE			
	Counts (% total)	Counts (% total)	Counts (% total)	P-value	OR (95% CI)	P-value
Diabetes						
Yes	78 (50.98%)	27 (17.65%)	5 (3.27%)			
No	38 (24.84%)	3 (1.96%)	2 (1.31%)			
Total	116 (75.82%)	30 (19.61%)	7 (4.58%)	0.0287*	4.11 (0.955-5.05)	0.0124* one VTE/no VTE
Outcome						
Discharged home	126 (65%)	25 (13%)	5 (3%)			
Referred	5 (3%)	2 (1%)	0 (0%)			
Deceased	20 (10%)	7 (4%)	5 (3%)			
Total	151 (77%)	34 (17%)	10 (5%)	0.0276*	5.1 (1.30-36.09)	0.0168* deceased/discharged

Furthermore, there was a statistically significant association between episodes of VTE and outcome with a p-value of 0.0276 and an OR of 5.1 for being deceased as opposed to being discharged from the hospital (CI = 1.30-36.09, p = 0.0168), as shown in Table 3.

## Discussion

Our study showed no association between gender and the number of VTE episodes. However, in their 2022 study, Wilcox et al. found that thrombosis and death were more common in men than women [21]. In our study, DM patients were four times more likely to develop at least one VTE episode when compared to those without DM. Calvisi et al. supported this data in their 2021 study, as they found that those with DM developed VTE more frequently than those without DM [22].

Patients who did not experience any VTE generally had shorter stays than those who developed one VTE episode. In addition, there was a significant difference between those who did not have VTE and the other two groups. This is in concordance with a 2022 study by Lee et al., which found a prolonged hospital and ICU stay in COVID-19 patients who developed VTE [23].

We found no significant difference between D-dimer, aPTT, PT, fibrinogen, INR, bilirubin, AST, ALT, creatinine, BUN, LDH, electrolytes, and the number of VTE episodes. Other studies have found an association between D-dimer, LDH, AST, BUN, PCT, and VTE [24,25]. A meta-analysis noted conflicting results regarding the significance of aPTT and PT in COVID-19 patients experiencing thromboembolic events [26].

Moreover, we found no significance for variables such as ferritin, CRP, and ESR. In contrast, a 2020 meta-analysis by Zeng et al. found that those in the non-severe COVID-19 group had lower CRP, ESR, and ferritin levels than those in the severe COVID-19 group [3]. There was no statistical significance between PCT levels for patients who had two or more VTE episodes and the other two groups. Conversely, a 2020 study by Wang et al. correlated PCT with the severity of COVID-19 patients in ICU and non-ICU settings [25].

The interplay among diabetes, COVID-19, and coagulopathy is complex and multifaceted. Diabetic patients typically exhibit a hypercoagulable state, which, when compounded with COVID-19 infection, significantly escalates the risk of thrombotic complications [27]. Our findings align with this notion, demonstrating that diabetic patients with COVID-19 are at an amplified risk of VTE development. This synergistic effect underscores the necessity for vigilant monitoring and potential prophylactic anticoagulation in this patient subgroup. This approach is also supported by the work of Zhang et al. (2020) who emphasized the importance of early detection and management of thrombotic complications in COVID-19 patients [1].

Regarding study limitations, the COVID-19 pandemic-related clinical settings imposed limitations that were tackled by utilizing convenience sampling, which provided a more sizeable data set at the cost of generalizability of the results. The medical records of NGHA were inconsistent in charting and lab workups. Furthermore, specific information regarding the medications given to patients during their admission was not provided in detail in the BestCare system from which our data was retrospectively collected. This, in part, is due to the coding schemes and charting having been oriented to patient care mainly, which makes extracting granular data such as medication use during the admission period very hard to acquire and sometimes provides inaccurate information as to when the medication was ordered and when it was administered. Because of this restriction, we were unable to evaluate how particular drugs affected the risk of VTE in the study participants. Our aim was also to consider cytokines and complement factors; however, they were not available in the patient laboratory workups. Additionally, it should be considered that thromboprophylaxis was given to every admitted patient. Finally, as more than half of our patients were within the 50-65-year age group, caution is needed before making conclusions about the association between age groups and the development of VTE.

## Conclusions

In our study, diabetes has been shown to be of interest when evaluating potential risk factors for VTE development in COVID-19 patients. Length of stay also plays a significant role in the severity of VTE in COVID-19 patients, which underscores the necessity for increased attentiveness and potentially revised treatment strategies for long-term hospitalized patients, especially those with diabetes.

Similar studies need to be conducted with a randomized sample on a national scale to better understand how the interactions between diabetes, duration of hospital stay, and our other studied variables across a broader patient demographic might influence the severity of VTE episodes in COVID-19 patients who have pre-existing DM and their potential utility as prognostic factors.
